# Eicosapentaenoic Acid (EPA) Induced Macrophages Activation through GPR120-Mediated Raf-ERK1/2-IKKβ-NF-κB p65 Signaling Pathways

**DOI:** 10.3390/nu9090937

**Published:** 2017-08-25

**Authors:** Lirong Han, Shumin Song, Yabing Niu, Meng Meng, Chunling Wang

**Affiliations:** Key Laboratory of Food Nutrition and Safety, Ministry of Education, College of food Engineering and Biotechnology, Tianjin University of Science and Technology, No. 29, 13th Avenue, Tianjin Economy Technological Development Area, Tianjin 300457, China; 15022275648@163.com (L.H.); duanjichao@mnchip.com (S.S.); coder_djc@163.com (Y.N.); 13512090919@126.com (M.M.)

**Keywords:** Eicosapentaenoic acid, immunomodulatory, NF-κB, RAW264.7 cells, Raf

## Abstract

*Objectives:* To investigate the immunomodulatory effect and molecular mechanisms of Eicosapentaenoic acid (EPA, a typical kind of n-3PUFAs) on RAW264.7 cells. *Methods:* A variety of research methods, including the RAW264.7 cells culture, cell proliferation assays, morphologic observations, measurements of NO production, cytokine assays, nuclear protein extractions, western blot analyses and NF-κB p65 immunofluorescence assays were used in this study. *Results:* The results showed that EPA could increase the proliferation index and enhance the release of nitric oxide (NO) and cytokines in RAW264.7 cells. Western blotting results revealed that the protein level of GPR120 increased significantly in RAW264.7 cells after EPA treatment. Meanwhile, EPA elevated the phosphorylation status of Raf, which may act as an upstream regulator of EPA-induced phosphorylated ERK1/2. In addition, the phosphorylated ERK1/2 may then promote IKKβ in endochylema and translocate the NF-κB p65 subunit into the nucleus, thus regulating the production of inducible nitric oxide synthase (iNOS) and cytokines. *Conclusions:* EPA (0.6–3.0 μmol) activates RAW264.7 cells through GPR120-mediated Raf-ERK1/2-IKKβ-NF-κB p65 signaling pathways.

## 1. Introduction

Polyunsaturated fatty acids (PUFAs) exhibit several favorable effects on cardiovascular diseases [1], autoimmune disorders and inflammatory disorders such as psoriasis [2], rheumatoid arthritis [3,4] and diabetes [5]. For more than 30 years, researchers have known that polyunsaturated fatty acids (PUFAs) can influence the immune system [6]. Eicosapentaenoic acid (EPA), a well-known dietary *n*-3 PUFA, is a long-chain PUFA that has 20 carbon atoms and 5 double bonds (20:5). Itis used as a food supplement with several beneficial effects, including the prevention of cardiovascular diseases, cancers and diabetes [7,8]. Although EPA and DHA intake as food or supplement effectively prevented cardiovascular events, cardiac deaths and coronary events in patients with high cardiovascular risk [9], it still has some controversy [10,11]. Some studies have suggested that dietary EPA can significantly affect human specific and nonspecific immunity [12]. In addition, research has shown that pre-treatment with 100 μmol of EPA decreases LPS-induced pro-inflammatory cytokine production by macrophages [13]. However, only a few studies have demonstrated that EPA induces the activation of macrophages. 

The immune system is a system of biological processes that protect against diseases. Disorders of the immune system can result in autoimmune diseases, inflammatory diseases and even cancer [14,15]. Macrophages occupy a unique niche in the immune system, as important target cells of some antitumor and immunomodulatory drugs [16]. In contrast to most other mouse-derived cell cultures, the macrophage-like cell line RAW264.7 [17] supports the replication of murine noroviruses and is widely used for this purpose [18]. Further, RAW264.7 cells are commonly accepted as a tool to investigate the molecular mechanisms of macrophages involved immuneregulation [19].

G protein-coupled receptors (GPCRs) are important signaling molecules for many aspects of cellular function. It was reported that five orphan receptors, GPR40, GPR41, GPR43, GPR84, and GPR120, can be activated by free fatty acids (FFAs). Long-chain FAs can activate GPR40 [20] and GPR120 [21]. GPR120 involved in homeostatic metabolic regulation in inflammatory and immune processes in vivo [22]. 

Macrophages defend against external pathogens by releasing cytotoxic and inflammatory molecules such as NO and also by secreting cytokines (IL-1β, IL-6, TNF-α and IFN-γ) [23]. These defense mechanisms are activated when various molecules trigger several different signaling pathways including mitogen-activated protein kinases (MAPKs) and nuclear factor kappa B (NF-κB) [24]. The Raf protein plays a pivotal role in regulating cell survival, cell cycle progression and cell differentiation [25]. ERK1/2, also called p44/p42 MAPK, also plays an essential role in a wide variety of cellular processes, including metabolism, cell proliferation and cell survival [26]. However, the relationship among them in the activation of macrophages has not been discussed.

The aim of this study was to elucidate the influence of a low dose of EPA induced activation in RAW264.7 cells. In this study, the mechanism responsible for a low dose of EPA activated RAW264.7 cells were examined. The protein expression of G-protein coupled cell membrane receptor (GPR120) was tested by Western blot to investigate the effect of EPA on RAW264.7 cells. Meanwhile, the production of NO, iNOS expression and the level of cytokines induced by EPA were measured. The expression of phosphorylated Raf, phosphorylated ERK1/2, IKKβ and NF-κB p65 were assessed after a low dose of EPA treatment. In addition, TAK632 (the inhibitor of Raf), PD98059 (the inhibitor of ERK1/2), and PDTC (the inhibitor of NF-κB) were used to investigate the relationship among them. The results suggested that a low dose of EPA up-regulated NO, iNOS and cytokine production via a GPR120-mediated Raf-ERK1/2-IKKβ-NF-κB p65 signaling pathway in RAW264.7 cells. These results will expand current knowledge of the mechanism of how EPA acts as a potent adjuvant and agent with immunomodulatory activity.

## 2. Materials and Methods

### 2.1. Materials

Eicosapentaenoic acid (EPA), 3-(4,5-Dimethylthiazol-2-yl)-2,5-diphenyltetrazolium bromide (MTT) and GPR120 antibody were purchased from Sigma (St. Louis, MO, USA). Penicillin-streptomycin solution, trypsin, phosphate buffer saline (PBS) and dimethyl sulfoxide side (DMSO) were purchased from Thermo (Beijing, China). PD98059 (inhibitor of ERK1/2) and pyrrolidine dithiocarbamic acid (PDTC, inhibitor of NF-κB p65) were also purchased from Sigma (St. Louis, MO, USA). The phospho-Raf, Raf, phospho-ERK1/2, ERK1/2, IKKβ, NF-κB p65, β-actin and horseradish peroxidase-conjugated secondary antibodies were purchased from Cell Signaling Technology (Danvers, MA, USA). The Raf inhibitor (TAK-632) was obtained from Selleck Chemicals (Houston, TX, USA). EPA standard was dissolved in dimethyl sulfoxide (DMSO) to 5 mg/mL, and then was added in the RPMI-1640 medium as the working solutions of EPA. All other chemicals were of the analytically purest grade available.

### 2.2. Cell Culture

RAW264.7 Abelson murine leukemia virus-induced tumor was obtained from The Cell Bank of Type Culture Collection of the Chinese Academy of Sciences (Shanghai, China), and RAW264.7 cells (5 × 10^5^ cells/mL) were cultured overnight in T-25 cell culture bottle (NEST Biotechnology Co., LTD., Wuxi, China) within a final volume of 4 mL RPMI-1640 medium (Solarbio, Beijing, China) supplemented with10% foetal bovine serum (Gibco BRL, Grand Island, NY, USA), 100 units/mL penicillin and 0.1 mg/mL streptomycin at 37 °C in a humidified chamber of 95% air and 5% CO_2_ atmosphere. 

### 2.3. Cell Proliferation Assay

The effect of EPA on the proliferation of RAW264.7 cells was determined by MTT assay [27]. RAW264.7 cells suspension (1 × 10^5^ cells/mL) was planted in 96-well micro titre plates (NEST Biotechnology Co., LTD., Wuxi, China) in a final volume of 100 μL RPMI-1640 medium supplemented with 10% foetal bovine serum, 100 units/mL penicillin and 0.1 mg/mL streptomycin at 37 °C in a 5% CO_2_ atmosphere. After 4 h, the RAW264.7 cells were washed twice with pre-warmed Hank’s Balanced Salt Solution and incubated in serum-free culture medium (200 μL/well) with 0.6–3.0 μmol EPA for 0–72 h. The control cells were treated with DMSO equaling within 3.0 μmol EPA group in RPMI-1640 medium (200 μL/well). After incubation, the cells were incubated with MTT solution (5 mg·mL^−1^, 20 μL/well) in medium for 4 h at 37 °C. Viable cells convert the MTT to formazan, which generates a blue-purple color after dissolving in 150 μL DMSO. The absorbance at 570 nm was the measured with a microplate reader (Model 680, Bio-Rad, Hercules, CA, USA). All tests were carried out in 6 independent experiments.

$$\begin{array}{cc} \mathrm{Cell}\;\mathrm{proliferation} & \left(\%\right)\\ & \;=[(\mathrm{absorbance}\;\mathrm{of}\;\mathrm{cells}\;\mathrm{treated}\;\mathrm{with}\;\mathrm{EPA}\\ & \;-\mathrm{absorbance}\;\mathrm{of}\;\mathrm{control}\;\mathrm{cells})/\mathrm{absorbance}\;\mathrm{of}\;\mathrm{control}\;\mathrm{cells}]\times 100\% \end{array}$$

### 2.4. Morphologic Observations

Acridine orange staining (Solarbio, Beijing, China) was carried out as previously described [28]. Briefly, RAW264.7 cells in the exponential growth phase were seeded in 24-well culture plates (NEST Biotechnology Co., LTD, Wuxi, China) at a final concentration of 5 × 10^5^ cells·well^−1^. After 4 h, the RAW264.7 cells were washed twice with pre-warmed Hank’s Balanced Salt Solution and incubated in serum-free culture medium with 0.6–3.0 μmol EPA for 24 h, and then stained with the staining solution which containing 100 μg·mL^−1^ acridine orange for 20 min at room temperature in the dark. Cells were observed under an inverted fluorescence microscope (Advanced Microscopy Group, Mill Creek, WA, USA) after the staining.

### 2.5. Measurement of NO Production

Nitrite accumulated in the culture medium was measured as an indicator of NO production based on the Griess reaction [29]. Briefly, RAW264.7 cells suspension (1 × 10^5^ cells/mL) was planted in 96-well microtitre plates (NEST Biotechnology Co., LTD., Wuxi, China) in a final volume of 100 μL RPMI-1640 medium supplemented with 10% foetal bovine serum, 100 units/mL penicillin and 0.1 mg/mL streptomycin at 37 °C in a 5% CO_2_ atmosphere. After 4 h, the RAW264.7 cells were washed twice with pre-warmed Hank’s Balanced Salt Solution incubated in serum-free culture medium (200 μL/well) with 0.6–3.0 μmol EPA for 24 h. The control cells were treated with DMSO equaling within 3.0 μmol EPA group in RPMI-1640 medium (200 μL/well). After incubation, 100 μL aliquots of the supernatant were distributed in a 96-well plate and then equal volumes of the Griess reaction solutions (1% sulfanilamide, 0.1% *N*-(1-naphthyl)-ethylenediamine dihydro-chloride in 2.5% phosphoric acid) were added. The reaction was allowed to proceed for 15 min at room temperature. The concentration of NO was calculated by extrapolating an NaNO_2_ standard curve.

### 2.6. Cytokine Assays

RAW264.7 cells were pre-incubated at 1 × 10^6^ cells·well^−1^ in a 12-well plate (NEST Biotechnology Co., LTD., Wuxi, China) for 4 h at 37 °C in a 5% CO_2_ and then were pretreated with 10 μmol PDTC (inhibitor of NF-κB) before being incubated with various concentrations of EPA (0–3.0 μmol) at 37 °C for 24 h. Levels of IL-1β, IL-6, TNF-α and IFN-γ in the culture supernatants were evaluated by using commercial ELISA kits (R&D Systems, Minneapolis, MN, USA) according to the manufacturer’s instructions. The absorbance was measured in an ELISA reader at 450 nm. The concentrations of cytokines were calculated according to the standard curve using each of the recombinant cytokines in the ELISA kits.

### 2.7. Nuclear Protein Extraction

Nuclear extracts were prepared by nuclei lysis in a high-salt buffer supplemented with protease and phosphatase inhibitors using a nuclear extraction kit (Panomics Inc., Fremont, CA, USA) according to the manufacturer’s protocol. Protein concentrations were quantified using the Bio-Rad protein assay (BCA Protein Assay Kit, Beyotime, Shanghai, China).

### 2.8. Western Blot Analysis

After various treatments as described previously, RAW264.7 cells were washed in cold PBS for 3 times and lysed with the Nuclear and Cytoplasmic Protein Extraction Kit (Beyotime, Shanghai, China). Protein concentrations were quantified with the BCA Protein Assay Kit using bovine serum albumin as a standard. All the primary antibodies were diluted with PBS for 1000 times (Cell Signaling Technology, Danvers, MA, USA). In the SDS-PAGE, the 5% concentrated gel and 12% separation gel were used for the western blotting analysis. In brief, cell lysates were subjected to SDS-PAGE and transferred to nitrocellulose membranes. The nitrocellulose membranes were then blocked in a TBST (Tris = 20 mM, pH 7.6, NaCl = 150 mM and Tween20 = 0.1%) containing 5% non-fatdrymilk powder (Bio-Rad. Co., Shanghai, China) and incubated with the indicated antibodies overnight at room temperature. The membranes were subsequently washed by TBST and incubated for 1 h at room temperature with the appropriate secondary anti- bodies conjugated with horseradish peroxidase (diluted with PBS for 100 times, Amersham Pharmacia Biotech, Piscataway, NJ, USA). The immune-reactive bands were detected with using an enhanced chemiluminescence (ECL) kit (Millipore Co., Billerica, MA, USA). Each test was performed in 6 independent experiments. Western blot analysis was carried out by the method as described previously [30].

### 2.9. Immunofluorescence Assay for NF-κB p65

Briefly, the cell suspension (1 × 10^5^ cells·well^−1^) was inoculated on coverslips that were partitioned previously into a 6-well plate (NEST Biotechnology Co., LTD., Wuxi, China). After 4 h, RAW264.7cells were treated with (0 or 2.4 μmol) EPA for 24 h. Cells were fixed with 3% formaldehyde in PBS for 20 min and washed with PBS for three times. Washed cells were permeabilized using 0.2% Triton X-100, and then blocked in 2% bovine serum albumin in PBS. Thereafter, cells were washed three times with PBS and incubated with the antibody NF-κB p65 (dilution 1:200) with 2% BSA in PBS at 37 °C for 1 h. The resulting cells were washed with PBS three times and incubated with fluorescein FITC-labeled polyclonal goat anti-rabbit IgG antibody (dilution 1:200) at 37 °C for 1 h, after which they were stained with propidium iodide (PI) (Sigma, St. Louis, MO, USA). The cells were, finally, scanned by laser scanning confocal microscopy (LSCM) after being washed again with PBS [31]. All images were acquired using the same intensity and photodetector gain.

### 2.10. Statistical Analysis

Results were expressed as the mean ±standard deviation (S.D.) of 6 independent experiments normalized to DMSO. Statistical analyses were performed using SPSS software (version 18.0; SPSS Inc., Chicago, IL, USA) to determine the significant differences. All values were analyzed by one-way analysis of variance (ANOVA) followed by a post hoc Tukey’s test for multiple comparisons. Values of *p* < 0.05 were considered statistically significant.

## 3. Result

### 3.1. EPA Increased the Proliferation Index of RAW264.7 Cells

**Figure 1:**
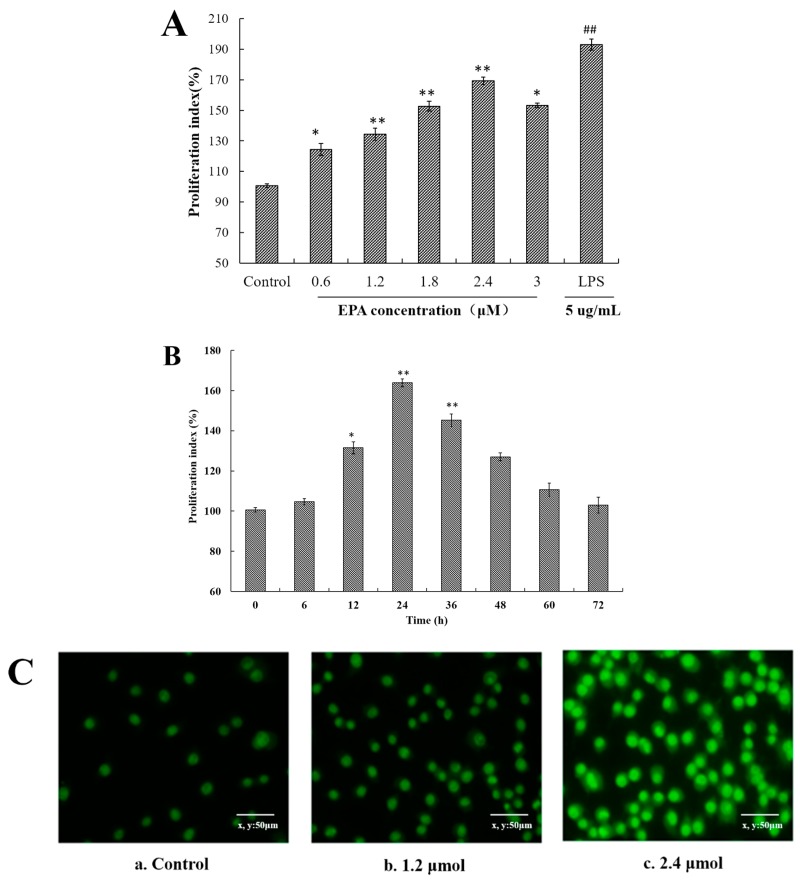
EPA induced cell proliferation index and morphological changes of RAW264.7 cells (**A**) Effect of EPA concentrations (0, 0.6, 1.2, 1.8, 2.4 and 3.0 μmol) on proliferation index in RAW264.7 cells; (**B**) Different culture times (0, 6, 12, 24, 36, 48, 60 and 72 h) of at a concentration of 2.4 μmol EPA on proliferation index in RAW264.7 cells. The control was the untreated cells which were incubated with DMSO and 0 μmol EPA. Each bar is representative of 6 independent experiments, and data were analyzed by ANOVA and Duncan’s multiple range tests. (Means ± SD, * *p* < 0.05 and ** *p* < 0.01 compared with the control; LPS group (positive control) ^##^ *p* < 0.01); (**C**) Cell morphology change was recorded by a fluorescence microscope (400×). **a**. Cells were treated with 0 μmol EPA for 24 h. **b**. Cells were treated with 1.2 μmol EPA for 24 h. **c**. Cells were treated with 2.4 μmol EPA for 24 h.

As shown in Figure 1A, EPA increased the cell proliferation at the concentrations of 0.6–3.0 μmol for 24 h in a dose-dependent manner in comparison with control. Meanwhile, EPA at 2.4 μmol also significantly enhanced the proliferation index of RAW264.7 cells in a time-dependent manner (Figure 1B). At the concentration of 2.4 μmol, the proliferation rate at 12 h was about 31.5% and at 24 h reached the maximal rate of about 63.9%. These results suggest that EPA can promote the RAW264.7 cells proliferation.

### 3.2. EPA Induced the Morphological Changes of RAW264.7 Cells

Once macrophages have been stimulated by an activator, some typical morphological changes are exhibited. Acridine orange is a basic stain whose planar structure is derived from anthracene. It shows a high affinity for nucleic acid, and intercalates into ds or binds electrostatically to ss nucleic acid emitting a green fluorescence at 530 nm and red fluorescence at 640 nm, respectively, under UV light [32].To further determine the morphological changes of RAW264.7 cells by EPA, cells were stained with acridine orange. In the control, RAW264.7 cells were normal in size and structure and the staining green fluorescence of those cells was weak (Figure 1(Ca)). In contrast to the control, the cells treated with 1.2 μmol EPA for 24 h showed a markedly stronger staining fluorescent intensity, and reached the peak value with 2.4 μmol EPA (Figure 1(Cb,c)). Meanwhile, the number of cells treated with 1.2–2.4 μmol EPA significantly increased in a dose-dependent manner. In the study above, the results suggested that EPA could promote the RAW264.7 cells proliferation (Figure 1C).

### 3.3. EPA Induced NO Production of RAW264.7 Cells

**Figure 2:**
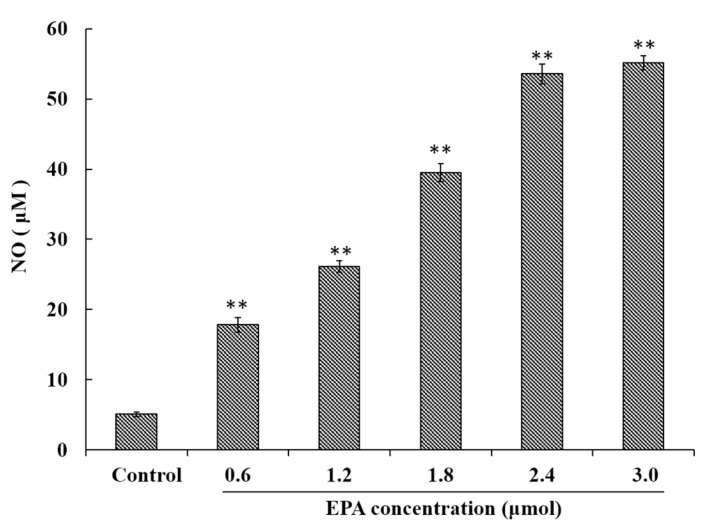
EPA regulated NO production of RAW264.7 cells. Cells were treated with DMSO (equivalent volume 3.0 μmol EPA, as control) or EPA at the various concentrations (1.2, 1.8, 2.4 and 3.0 μmol) for 24 h. (Means ± SD, ** *p* < 0.01 compared with the control). Each bar is representative of 6 independent experiments, and data were analyzed by ANOVA and Duncan’s multiple range tests.

NO has been demonstrated to play an important role in a variety of physiological effects, host defenses and immunity responses [33]. To investigate whether or not EPA was capable of inducing NO production in macrophages, RAW264.7 cells were treated with EPA (0–3.0 μmol) for 24 h. As shown in Figure 2, a minimum amount of NO was released in the control, though cells treated with EPA at concentrations from 0 to 3.0 μmol showed significantly (*p* < 0.01) higher NO production in a dose-dependent manner. These results indicate that EPA can enhance immune-stimulatory activity such as the release of NO.

### 3.4. Effect of EPA on GPR120 Protein Expression

**Figure 3:**
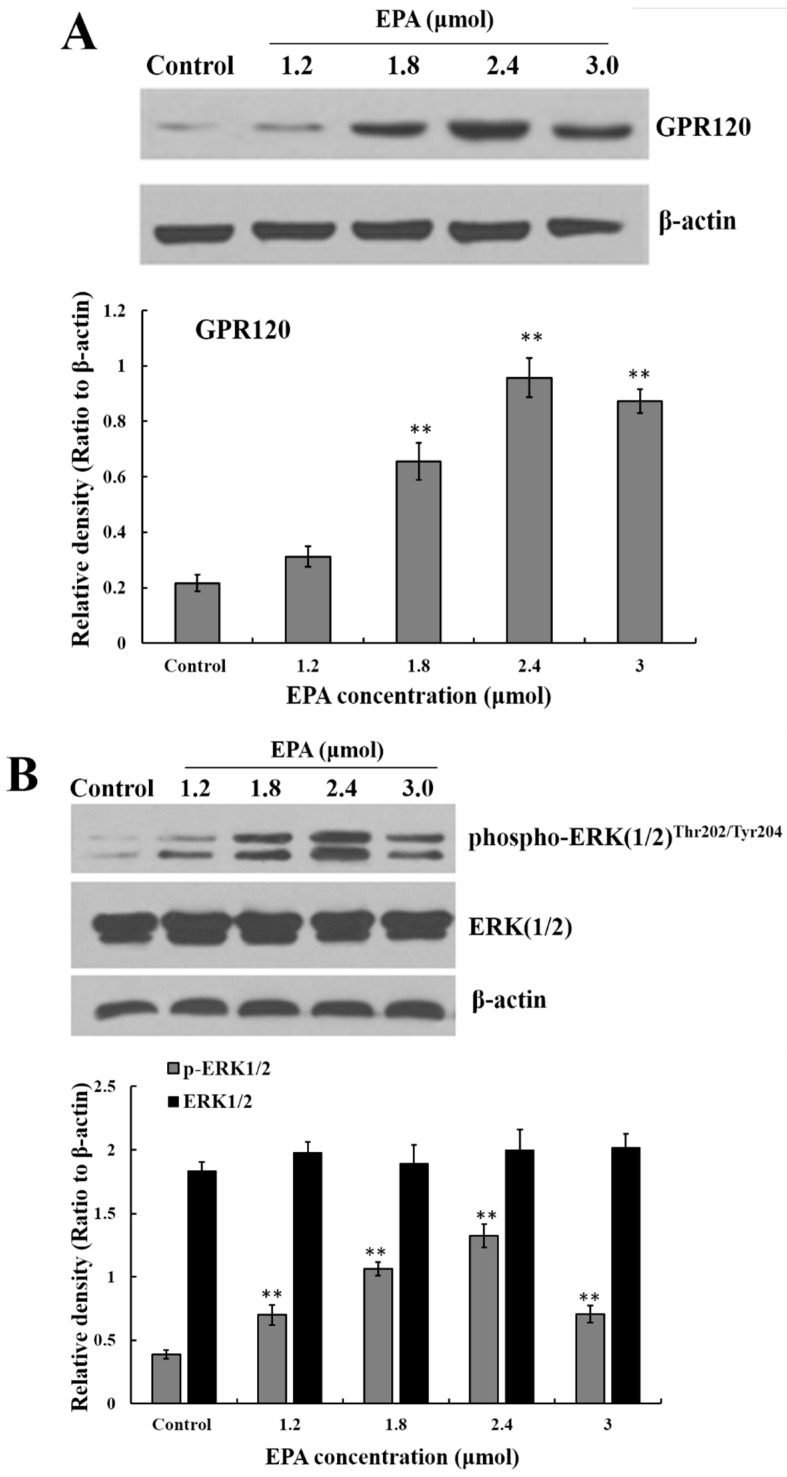

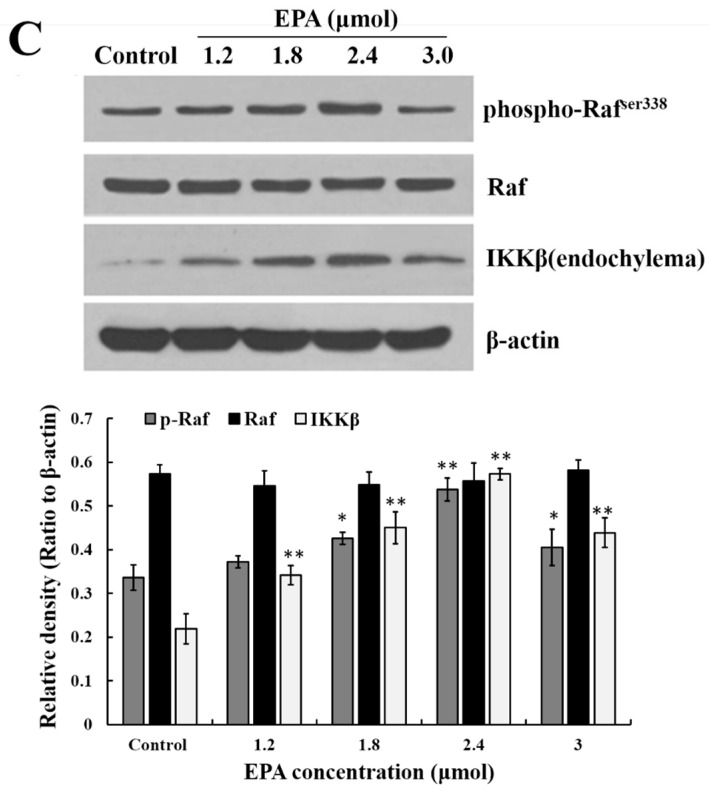
Measurement of GPR120, ERK, Raf and IKKβ. (**A**) Effect of EPA on the protein expression of GPR120 in RAW264.7 cells; (**B**) Effect of EPA on the protein expression of ERK1/2 in RAW264.7 cells; (**C**) Effect of EPA on the protein expression of Raf and IKKβ in RAW264.7 cells. Cells were treated with DMSO (equivalent volume 3.0 μM EPA, as control) or various concentrations (1.2, 1.8, 2.4 and 3.0 μmol) of EPA for 24 h. The expression of protein was analyzed by Western blot. The β-actin was used as an equal loading control (means ± SD, * *p* < 0.05 and ** *p* < 0.01 compared with the control). Each bar is representative of 6 independent experiments, and data were analyzed by ANOVA and Duncan’s multiple range tests.

GPR120 is a G-protein coupled cell membrane receptor expressed on macrophages which can bind with long-chain fatty acids. A marked increase in GPR120 protein level was detected in RAW264.7 cells after treatment with EPA relative to the control, and the protein expression of GPR120 reached peak value at the concentration of 2.4 μmol EPA (Figure 3A).

### 3.5. Measurement of ERK, Raf and IKKβ

The stimulation of GPR120 by FFAs may result in activation of the ERK cascade [21]. Recent studies have shown that the Raf protein can mediate diverse biological functions such as cell growth, cell survival and cell differentiation [34]. Activation of inhibitor kappa B kinase (IKK) stimulates the translocation and transactivation of transcription factor nuclear factor-kappa B (NF-κB), which then induces the expression of certain inflammatory genes [35]. As shown in Figure 3B,C, the level of phosphorylated ERK1/2, phosphorylated Raf and IKKβ in endochylema increased in a dose-dependent manner and reached their maximum value at the concentration of 2.4 μmol EPA. 

### 3.6. Relationship between ERK1/2 and Raf, IKKβ

**Figure 4:**
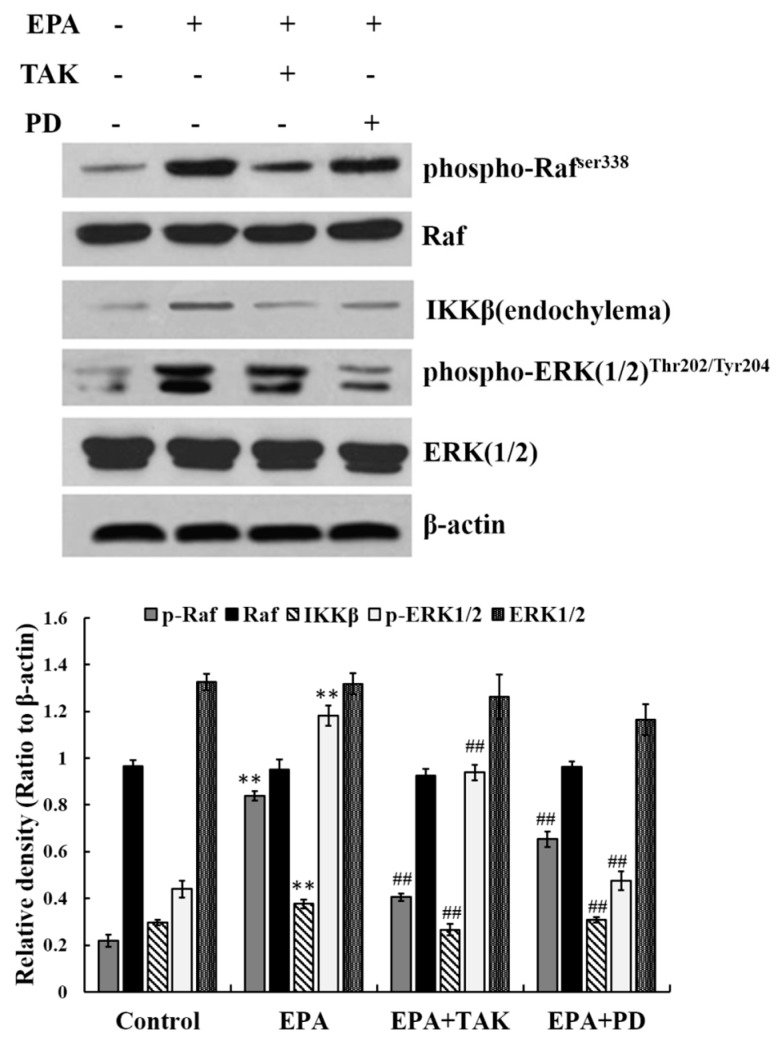
The relationship among ERK1/2, Raf and IKKβ. **C**ells were treated with DMSO (equivalent volume 3.0 μM EPA, as conrtrol) or 2.4μmolof EPA for 24 h in the presence or absence of 10 μmol PD or 25 nmol TAK. The expression of protein was analyzed by Western blot. The β-actin was used as an equal loading control. Each bar is representative of 6 independent experiments, and data were analyzed by ANOVA and Duncan’s multiple range tests (means ± SD, ** *p* < 0.01 compared with the control; ^##^ *p* < 0.01 compared with EPA group).

The results above demonstrate that phosphorylated ERK1/2, phosphorylated Raf and IKKβ in endochylema are involved in EPA- induced RAW264.7 cell activation. In order to further assess the relationship, cells were pre-incubated with either PD98059 (PD, an inhibitor of ERK1/2, at 10 μmol) or TAK 632 (TAK, an inhibitor of Raf, at 25 nmol) prior to adding EPA at a concentration of 2.4 μmol. The expression of phosphorylated Raf, phosphorylated ERK1/2 and IKKβ was then estimated. The level of Raf phosphorylation was not reduced by PD98069 (inhibitor of ERK1/2), which indicates that Raf phosphorylation is not dependent on ERK1/2. The increasing level of phosphorylated ERK1/2 was effectively blocked not only by its own inhibitor PD98069, but also by TAK632 in RAW264.7 cells. Simultaneously, the expression of IKKβ in cytoplasm was significantly decreased by PD98059 (inhibitor of ERK1/2) (Figure 4). The results indicate that IKKβ expression in cytoplasm is dependent on ERK1/2 phosphorylation, and also leads to the hypothesis that Raf may act as an upstream regulator of EPA induced ERK1/2 phosphorylation in RAW264.7 cells.

### 3.7. Effect of EPA on the Expression of NF-κB p65

**Figure 5:**
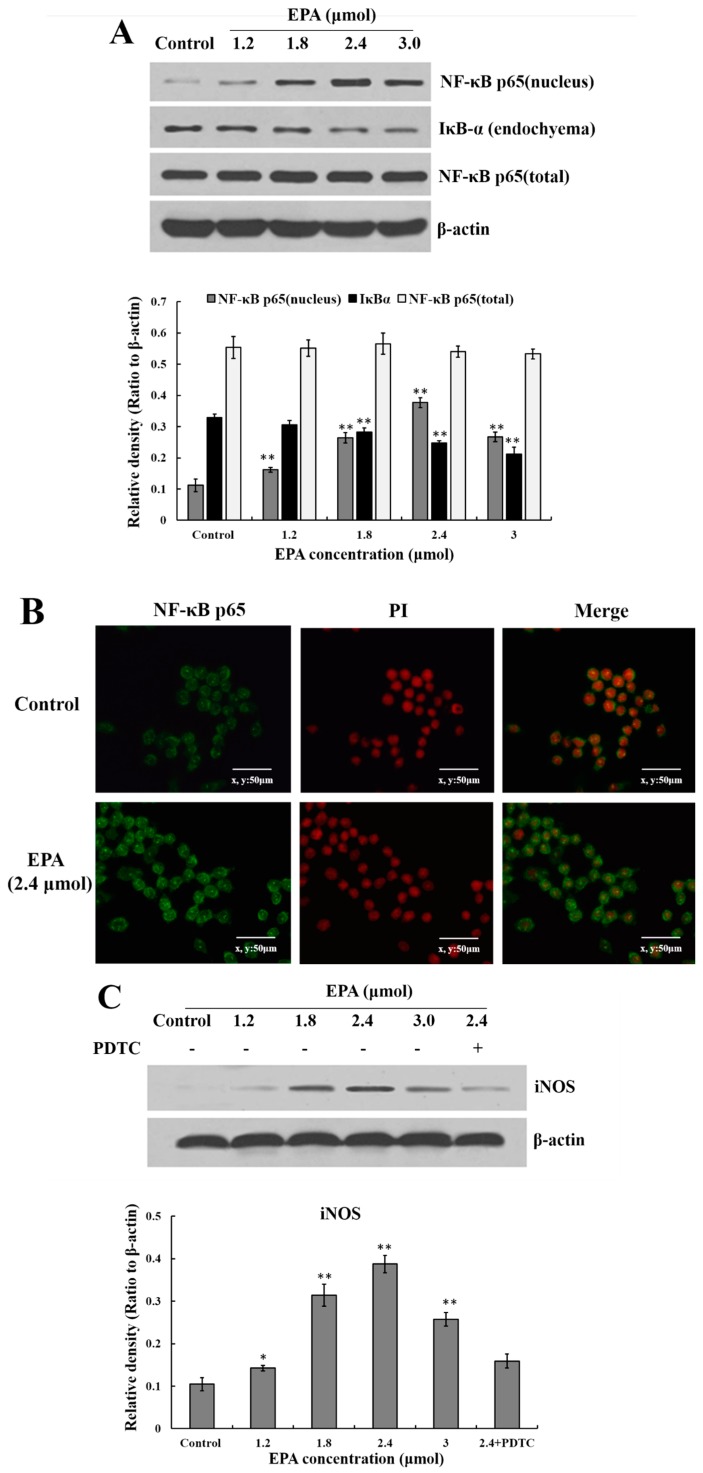

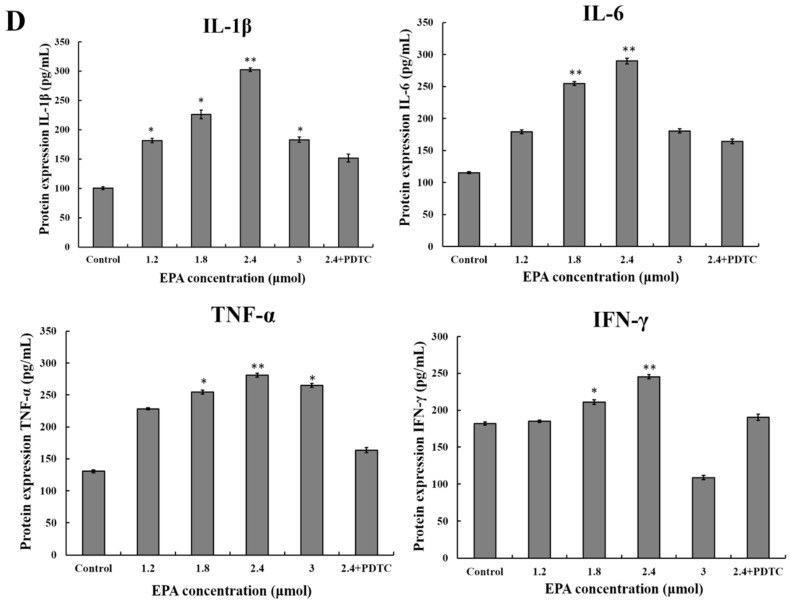
Effect of EPA on NF-κB p65, iNOS and cytokines protein levels of in RAW264.7 cells. (**A**) Effect of EPA on the protein expression level of NF-κB p65 and IκB-α in RAW264.7 cells. The expression of protein was analyzed by Western blot. The β-actin was used as an equal loading control; (**B**) Immunofluorescence staining demonstrating the effects of DMSO (equivalent volume 3.0 μmol EPA, as control) or EPA (3.0 μmol) on subcellular localization of NF-κB p65 in RAW264.7 cells. Visualization of the NF-κB p65 fluorescence was recorded by using a LSCM, bar = 50 µm (×400); (**C**) EPA regulated the protein expression of iNOS; (**D**) EPA regulated cytokines protein levels of RAW264.7 cells. Cells were treated with DMSO (equivalent volume 3.0 μmol EPA, as control) or EPA at the various concentrations (1.2, 1.8, 2.4 and 3.0 μmol) for 24 h in the presence or absence of pretreated 10 μM PDTC. (Means ± SD, * *p* < 0.05 and ** *p* < 0.01 compared with the control). Each bar is representative of 6 independent experiments, and data were analyzed by ANOVA and Duncan’s multiple range tests.

NF-κB is an important transcription factor in the regulation of pro-inflammatory mediators in activated macrophages. IκB is a protein bound to dimers of NF-κB, which retains these transcription factors in the cytoplasm. An activation of IκB kinase (IKK) leads to the phosphorylation of IκB and its degradation which allow nuclear localization of NF-κB [36,37]. To investigate whether or not EPA activates the NF-κB signaling pathway, the nuclear level of NF-κB p65 and IκB-αwere analyzed by Western blot. As shown in Figure 5A, IκBα proteolytically degraded and free NF-κB p65 translocated into the nucleus. Meanwhile, under the LSCM, the intensity of NF-κB p65 fluorescence in the nucleus treated by EPA (2.4 μmol) was significantly stronger than that of the control (Figure 5B). The results above indicate that EPA can induce NF-κB activation in RAW264.7 cells. 

### 3.8. EPA Regulated the Protein Expression of iNOS and Cytokines

The current study suggests that EPA (0.6–3.0 μmol) can promote NO production. In order to further evaluate whether or not the improvement of NO synthesis by EPA at concentration from 0 to 3.0 μmol was due to the enhancement of iNOS expression, cells were incubated with EPA (0–3.0 μmol) for 24 h in either the presence or absence of pretreated 10μmol PDTC (inhibitor of NF-κB). The protein expression of iNOS was then measured by western blot. As shown in Figure 5C, the level of iNOS increased in a dose-dependent manner in RAW264.7 cells treated with EPA (1.2–2.4 μmol), while PDTC pretreatment can suppress EPA-induced iNOS production. Because of the prominent role of cytokines in the maturation and function of macrophages, the potential for EPA to regulate the expression of these mediators in RAW264.7 cells were examined. As shown in Figure 5D, the addition of EPA resulted in an increase in IL-1β, IL-6, IFN-γ and TNF-α protein levels in a dose-dependent manner. Additionally, the protein levels of all cytokines decreased in the presence of PDTC. These results suggest that the activation of NF-κB is one of the essential factors for iNOS production and cytokine expression in RAW264.7 cells treated with EPA, and further confirm that EPA can promote the activation of RAW264.7 cells.

## 4. Discussion

PUFAs have been shown to have a large variety of biological effects [38,39]. In recent years, tremendous research efforts have been put into establishing the different effects of various fatty acid families and individual fatty acids on a range of immune responses measured in vitro, ex vivo and in vivo, in an attempt to isolating mechanisms to prevent and/or treat diseases or conditions with an immunological basis [40]. Although efforts have been put in this research area, the immunomodulatory activity of PUFAs has not been clearly explained. Like the molecular mechanism responsible for EPA activated immunocytes such as macrophages remains to be clarified. The purpose for the present study is to investigate the activation of EPA on RAW264.7 cells and its subsequent intracellular signaling pathways. 

In the present study, the immunomodulatory effect and molecular mechanisms of EPA (0.6–3.0 μmol) on RAW264.7 cells were investigated. So far, the relative study has showed that the concentration of EPA in serum phospholipids was 0.9 mg/100 mL (about 0.8% of FAME) in persons taking chicken fed soybean oil supplement [41]. Due to the fact that our experiments were performed in vitro, it was unable to confirm the relationship between the active concentration of EPA used in the experiments and the serum concentrations of people taking varying levels of EPA containing foods. It would be imperative to investigate the effect of EPA in vivo in the future study.

Macrophage activation is one of the most important events in the immune response system [42]. NO is also related to the cytolysis function of macrophages against a variety of antigens and cytokines are key signaling molecules of the immune system. In addition, activated macrophages participate in immune responses by releasing cytokines such as IL-1β, IL-6, IFN-γ and TNF-α [43]. Our results indicate that a low dose of EPA can effectively enhance the proliferation index of RAW264.7 macrophages and increase the production of NO and cytokines in RAW264.7 cells, which verified that EPA could activate macrophages and participate in immune responses. 

GPR120 is a functional ω-3 FA receptor/sensor and mediates potent insulin sensitizing and antidiabetic effects in vivo by repressing macrophage-induced tissue inflammation. According to results, after EPA treatment at concentration of 1.2–3.0 μmol, an up-regulated GPR120 protein expression was found in RAW264.7 cells suggesting that GPR120 might be the one signal that ultimately results in intracellular signaling pathways for downstream NF-κB p65 activation thus leading to the production of iNOS. The result also revealed that GPR120 was the receptor of EPA on the RAW264.7 cells surface, which is consistent with the literature reports. 

MAPKs play a vital role in cell proliferation, migration and mobility [44]. ERK1/2 is a major member of the MAPK family [45]. Furthermore, the Raf protein also plays a critical role in the MAPKs pathway [46]. The Raf proteins are located upstream of MEK and activated Raf phosphorylates MAPK kinase 1/2 (MEK 1/2), which in turn phosphorylates and activates extracellular signal-regulated kinase 1/2 (ERK 1/2) [47]. The current study suggests that EPA induces the phosphorylation of Raf, the phosphorylation of ERK1/2 and increases the IKKβin RAW264.7 cells, whereas pretreatment with TAK-632 and PD98059 clearly antagonizes the up-regulated expression of ERK1/2 phosphorylation and IKKβ in endochylema. This effect indicates that IKKβ expression is dependent on the ERK1/2 phosphorylation, and phosphorylated Raf may act as an upstream regulator of EPA induced ERK1/2 phosphorylation in RAW264.7 cells. 

The activation of NF-κB is regulated by cellular kinases, including MAPK [48]. NF-κB is an important transcription factor that regulates a number of genes, including iNOS, TNF-α andIL-6which are important for immunity and inflammation [49]. NF-κB is generally inactive in the cytoplasm and is involved in the non-covalent binding form of the p50–p65-inhibitor κB (IκB) complex. When activation signals of NF-κB are received, serine residues in IκB are phosphorylated and dissociated from NF-κB, and are then transferred to the nucleus as an activated transcription factor [50]. After IκB degradation, the binding sites of the p50–p65 dimer are exposed to combine with the IκB motif. Then the NF-κB p65 subunit transfers from the cytoplasm to the nucleus, which in turn presents potent activity. Furthermore, because of the major role of the transcription factor in immunity, the NF-κB pathway has drawn much attention and has become a central target of drugs used to treat cancer.

When cells were stimulated by EPA, NF-κB was translocated into the nucleus, which in turn modulated gene expressions. Total NF-κB (p65) and IκB were detected by western blotting. As shown in Figure 5A,B, the level of total NF-κB (p65) nearly unchanged after treatment with EPA, whereas that of NF-κB in nucleus displayed an increased trend. These results show that IκBα proteolytic degradation and free NF-κB p65 translocation into the nucleus are both induced by EPA in RAW264.7 cells, which indicated that the activation of RAW264.7 cells by EPA were associated with the IκB-NF-κB signaling pathway.

## 5. Conclusions

**Figure 6:**
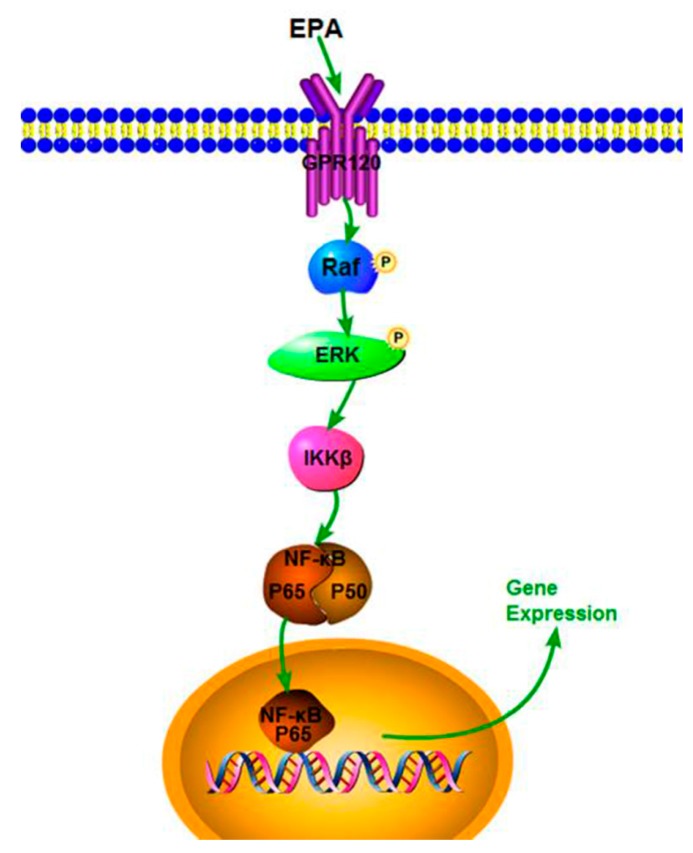
The sketch map of the RAW264.7 cells proliferation induced by EPA.

Our study demonstrates that EPA (1.2–3.0 μmol) has potent immune-stimulatory activity in RAW264.7 cells. EPA can also effectively improve NO production, iNOS expression and cytokine levels, which might be due to the activation of the NF-κB p65 signal pathway. In addition, EPA promoted the proliferation index of RAW264.7 cells through GPR120-mediated Raf, ERK1/2 and NF-κB signaling pathways. A speculated schematic diagram of EPA-induced RAW264.7 cell activation is depicted in Figure 6. These results expand current knowledge of the mechanism of how EPA acts as a potent adjuvant and agent with immunomodulatory activity. The current study only used the RAW264.7 cells to investigate the immune-stimulatory activity of EPA in vitro. In the next study, we will explore the immune-stimulatory activity of EPA on other cell lines, such as human cells such as THP-1 cells or primary monocyte derived macrophages, or the effect of EPA in vivo.
